# Antigliotic guiding regenerative gel: a bioactive hydrogel platform for peripheral nerve and spinal cord repair

**DOI:** 10.3389/fsurg.2026.1902070

**Published:** 2026-08-10

**Authors:** Shimon Rochkind, Mara Almog, Arie Goldlust

**Affiliations:** 1 Faculty of Medicine, Tel-Aviv University, Tel-Aviv, Israel; 2 Microsurgical Center for Peripheral Nerve Reconstruction, Assuta Medical Center, Tel-Aviv, Israel; 3 Maxonis Ltd, Ness Ziona, Israel

**Keywords:** antigliotic guiding regenerative gel (AGRG), glatiramer acetate, hyaluronic acid, hydrogel, laminin peptide, LDP-916, nerve regeneration

## Abstract

Peripheral nerve injury (PNI) and spinal cord injury (SCI) impose a major burden of traumatic neurological disability worldwide. Autologous nerve grafting, the current gold standard, achieves meaningful recovery in only 81.6% of repairs (2023 meta-analysis, *n* = 1,559), while hollow nerve graft conduits achieve only 62.2% (*p* < 0.05), with performance declining sharply beyond 20–30 mm gaps. Passive scaffolds cannot counter the inhibitory post-injury microenvironment of reactive gliosis, chondroitin sulfate proteoglycan (CSPG) deposition, oxidative stress, and myelin-associated inhibitors, which actively collapse regenerating axon growth cones. This review traces the conceptual and developmental logic of a family of bioactive hydrogels, “neurogels”, designed as active modulators of the regenerative microenvironment rather than passive bridges. We organize the review around the scientific problem: the biological barriers that passive scaffolds cannot address; the design rationale for a hyaluronic acid (HA)-based neurogel bearing a biomimetic laminin-derived peptide (LDP-916), an antioxidant, and an antigliotic immunomodulator; the formulation evolution from the original Guiding Regenerative Gel (GRG; HA + LDP-916 + recombinant superoxide dismutase 1, SOD1) to the current Antigliotic Guiding Regenerative Gel (AGRG; HA + LDP-916 + DL-α-tocopherol + glatiramer acetate); and the preclinical evidence base. An *in vitro* ^35^S-glycosaminoglycan candidate-selection screen in activated astrocytes identified several antigliotic agents; translational barriers of the top-ranked biologics (bacterial origin and thermolability of chondroitinase ABC; blood-brain barrier impermeability of anti-NogoA antibody) motivated selection of glatiramer acetate, the active copolymer of Copaxone (a clinically validated multiple sclerosis drug), and replacement of SOD1 by DL-α-tocopherol for manufacturing and stability reasons. AGRG increased neurite length by approximately 78% *in vitro* relative to control, showed electrophysiological performance comparable to or exceeding autograft in a 25 mm chronic rabbit PNI model at 40 weeks, and promoted myelinated axon regrowth in acute and chronic rat SCI. A recently completed 15 mm rat sciatic critical-gap study comparing AGRG to autograft provides supportive internal data; because this dataset has not yet completed independent peer review, its findings are described in general terms and are not used as a basis for definitive efficacy claims. Regulatory-enabling studies are being pursued by Maxonis Ltd. in preparation for an FDA pre-IND interaction.

## 1 Introduction

### 1.1 Epidemiology and clinical burden of peripheral nerve and spinal cord injury

Peripheral nerve injury (PNI) is among the most consequential yet underappreciated traumatic conditions in modern surgery. Occurring in approximately 2.8% of all trauma patients (1), PNI results from a diverse array of mechanisms including motor vehicle collisions, industrial accidents, military-related blast and penetrating wounds, and iatrogenic injury during surgical procedures. The annual global incidence is estimated at 13.9 cases per 100,000 inhabitants per year (1), translating to approximately 300,000 new cases per year worldwide (1). In the United States alone, approximately 50,000 nerve grafting procedures are performed annually, incurring expenditures estimated at seven billion US dollars (1). Beyond the direct economic costs, PNI imposes profound personal burdens: partial or complete loss of sensory and motor function, intractable neuropathic pain, muscle atrophy, and progressive long-term disability that demands sustained rehabilitation and frequently prevents return to productive employment.

Spinal cord injury (SCI) represents an analogous but even more devastating condition within the continuum of neurotraumatic disorders. Approximately 17,810 new SCI cases are reported each year in the United States alone, with lifetime care costs exceeding $600,000 per patient (2). The global incidence of SCI is estimated at 10.5 per 100,000 persons annually, with approximately 768,473 new cases worldwide each year (3). These figures reflect not only direct medical expenditure but the profound disability burden imposed over decades of life expectancy with impaired function. The median age at injury is 31.7 years (4), meaning the disease predominantly strikes working-age adults, compounding the socioeconomic impact across decades of disability. Unlike peripheral nerves, the central nervous system possesses a markedly limited intrinsic regenerative capacity, and the injury microenvironment, dominated by reactive gliosis, CSPG deposition, and myelin-associated inhibitors, actively suppresses the axonal regrowth that does occur. Section 2 examines this inhibitory microenvironment in mechanistic detail, since it is the central biological problem that motivates the entire design rationale of the neurogel platform described in this review.

### 1.2 Current standard of care and its limitations

The surgical treatment of PNI with significant tissue loss depends on the gap length and clinical context. For short gaps that permit tension-free coaptation, primary neurorrhaphy remains appropriate. When a gap exists, three principal reconstructive strategies are available: autologous nerve grafting, processed nerve allografts, and tubulization with a synthetic or biological nerve graft conduit (NGC).

Autologous nerve grafting (ANG) has been the gold standard for peripheral nerve reconstruction for decades. ANG provides a naturally biomimetic scaffold incorporating basal lamina microtubes, viable Schwann cells, neurotrophic factors, and native extracellular matrix (ECM) architecture, collectively creating a permissive, actively guidance-providing regenerative highway (5). Despite these biological advantages, ANG carries inherent limitations: donor-site morbidity including sensory deficit and painful neuroma at the harvest site, limited graft availability for large reconstructions, and fascicular diameter mismatch (1, 5).

Currently, 11 FDA-approved NGCs are commercially available for PNI treatment, encompassing collagen-based devices (NeuraGen, NeuraGen 3D), synthetic polymer NGCs (Neurotube/PGA, Neurolac/PCL, SaluBridge/PVA), and processed decellularized nerve allograft (Avance® Nerve Graft, AxoGen) (6, 7). These devices avoid donor-site morbidity and offer surgical simplicity. The processed allograft (Avance® Nerve Graft) preserves ECM architecture while removing immunogenic cellular components, achieving meaningful recovery rates that approach those of autograft for defects up to 70 mm without donor-site complications (8).

However, the most rigorous comparative evidence, the 2023 systematic review and meta-analysis by Lans and colleagues (8), encompassing 1,559 repairs across 35 clinical studies spanning gap lengths from >5 to 70 mm, reveals a clear performance hierarchy. Meaningful recovery (defined as ≥S3/M3 by the Medical Research Council classification) was achieved in 87.1% of processed allograft repairs, 81.6% of autograft repairs, and only 62.2% of hollow NGC repairs (*p* < 0.05) (8). In long-gap repairs (>30 mm), NGC performance deteriorates further, while allografts maintain meaningful recovery approaching ANG levels. Hollow NGC demonstrate acceptable outcomes only in short sensory gaps (≤20–30 mm), with rapid performance decline at greater lengths attributable to luminal collapse, failure to maintain a stable fibrin scaffold, and insufficient Schwann cell migration across the gap (6, 8).

For spinal cord injury, treatment options remain fundamentally limited. Surgical decompression and stabilization represent acute interventions that prevent secondary injury progression but do not restore function. Methylprednisolone administration in the acute setting remains controversial, with current guidelines not recommending it as a standard of care due to inconsistent efficacy and significant adverse effects. Stem cell transplantation strategies have generated substantial research interest but have not yet achieved definitive clinical proof of efficacy, and growth factor delivery by injection or cell-based systems has demonstrated limited benefit in isolation. Recent multidisciplinary clinical practice recommendations for the surgical management of acute traumatic SCI emphasize early decompression and a structured, evidence-graded approach to surgical timing, underscoring that even contemporary best surgical practice does not resolve the underlying biological barrier to regeneration (9). The fundamental challenge, countering the glial scar's inhibitory molecular environment while simultaneously providing a physical bridge for regenerating axons, demands a solution that simultaneously addresses structure, biology, and biochemistry.

### 1.3 Aims and scope of this review

This review traces the conceptual and developmental history of the GRG/AGRG family of bioactive hydrogels, GRG (Guiding Regenerative Gel), the original formulation, and AGRG (Antigliotic Guiding Regenerative Gel), its current, bioactively enriched successor. Rather than proceeding strictly chronologically, the review is organized around the underlying scientific logic: Section 2 characterizes the biological barriers to nerve regeneration that motivate an active, rather than passive, scaffold design. Section 3 develops the design rationale for a bioactive neurogel component by component. Section 4 traces the formulation evolution from early HA hydrogels through GRG to AGRG. Section 5 presents the preclinical evidence base in PNI and SCI models. Section 6 situates AGRG within the broader landscape of competing and complementary nerve repair technologies, including alternative hydrogel platforms and cell-based strategies. Section 7 discusses translational status and open scientific questions, including several raised directly by peer review of this manuscript. The aim throughout is to give the reader a mechanistic understanding not only of what AGRG does, but of why each design decision was made, equipping clinicians, researchers, and regulatory scientists with the intellectual framework needed to evaluate this technology against the alternatives.

This review is dedicated to the memory of Professor Zvi Nevo, co-inventor of GRG and AGRG, whose foundational contributions to proteoglycan biochemistry and nerve regeneration biology made this body of work possible.

## 2 Biological barriers to nerve regeneration

### 2.1 The inhibitory post-injury microenvironment

Following both peripheral nerve and spinal cord injury, the local milieu is dominated by reactive gliosis, the pathological activation of astrocytes and Schwann cells, which produces large quantities of chondroitin sulfate proteoglycans (CSPGs). Spinal cord injury triggers an immediate and sustained cascade of molecular and cellular events that collectively constitute the glial scar, one of the principal barriers to axonal regeneration in the adult central nervous system (CNS) (10–12). Within hours of injury, blood-brain and blood-spinal cord barrier disruption leads to infiltration of macrophages, neutrophils, and leukocytes. Within days to weeks, reactive astrocytes, normally homeostatic cells essential for synaptic support, blood-brain barrier maintenance, and ion buffering, undergo a dramatic phenotypic transformation termed reactive astrogliosis, characterized by cellular hypertrophy, process extension, and markedly upregulated synthesis of inhibitory ECM molecules (10, 13). The resulting glial scar serves a short-term protective function by limiting the spread of cytotoxic cellular debris and containing the inflammatory response, but in the chronic phase it creates a formidable physical and biochemical barrier to axonal regeneration (13, 14).

The principal molecular constituents of the glial scar that mediate regenerative inhibition are the CSPGs: neurocan, phosphacan, brevican, versican, NG2 (also designated CSPG4), and aggrecan (10, 11, 15, 16). These large, heavily glycosylated molecules are anchored in the pericellular and extracellular matrix around reactive astrocytes and other non-neuronal cells, where their negatively charged chondroitin sulfate glycosaminoglycan (GAG) side chains create steric and charge-based barriers to axonal growth cone advance (11, 17). At the molecular level, CSPGs exert their inhibitory effects through high-affinity interactions with two neuronal transmembrane receptor tyrosine phosphatases: LAR (leukocyte common antigen-related phosphatase) and PTP*σ* (protein tyrosine phosphatase sigma) (18, 19). CSPG binding to LAR and PTP*σ* activates intracellular signaling cascades, including the RhoA/ROCK pathway, that cause growth cone collapse and axonal retraction (18–20). These CSPGs are upregulated in the glial scar to levels many-fold above baseline, creating a potent repulsive field that persists for months to years after injury (15, 21).

Additional inhibitory constituents of the glial scar include remnants of myelin and oligodendrocyte-associated inhibitors, particularly Nogo-A (also called reticulon-4A) and its receptor complex (22–24); myelin-associated glycoprotein (MAG) (25); oligodendrocyte-myelin glycoprotein (OMgp); and collagenous ECM residues that accumulate in the fibrotic component of the scar, further impeding axonal penetration (26, 27). Nogo-A binds the Nogo receptor (NgR) expressed on axon growth cones, triggering intracellular signaling, again mediated largely through RhoA/ROCK, that inhibits regenerative growth (24, 28). The convergence of CSPG-mediated, myelin-associated, and structural barriers at the SCI site creates a deeply hostile environment that explains why even axons with intact intrinsic growth capacity fail to traverse the lesion in the adult CNS.

The temporal evolution of the glial scar has important implications for therapeutic targeting. In the hyperacute phase (0–24 h), infiltrating neutrophils and resident microglia are the dominant cellular actors, releasing pro-inflammatory cytokines (IL-1β, TNF-α, IL-6) and reactive oxygen species that amplify secondary axonal injury. In the subacute phase (days 1–2 weeks), reactive astrocytes begin to hypertrophy, upregulate glial fibrillary acidic protein (GFAP), and migrate toward the injury epicenter, while macrophages phagocytose myelin debris and begin producing TGF-β1 and TGF-β2, the principal cytokine drivers of CSPG synthesis by astrocytes (28, 29). CSPG deposition peaks during weeks 2–4 after injury, when the dense, inhibitory pericellular matrix surrounding reactive astrocytes reaches its maximum concentration (30, 31). In the chronic phase (months to years), the glial scar becomes a stable, cross-linked ECM barrier in which neurocan, versican, phosphacan, and NG2 persist at high levels, with negligible spontaneous degradation by endogenous matrix metalloproteinases (32, 33). It is this chronic, consolidated glial scar that is most clinically relevant, since the majority of patients with established SCI present in the chronic phase when treatment is sought.

While the glial scar is predominantly described in the context of CNS injury, the peripheral nervous system is not immune to CSPG-mediated inhibition. Following peripheral nerve injury, denervated Schwann cells, normally among the most important pro-regenerative cells in the PNS, capable of secreting neurotrophic factors and providing basal lamina guidance, also upregulate CSPG production (32, 33). Kuffler and colleagues demonstrated that Schwann cell-derived CSPGs inhibit dorsal root ganglion neuron neurite outgrowth through a soma-mediated mechanism, reducing the efficacy of peripheral nerve regeneration even in the permissive PNS environment (32). This observation is especially relevant for the chronic rabbit PNI model described in Section 5, where a nine-week delay between injury and reconstruction creates a Schwann cell environment approximating the chronically denervated, CSPG-rich state seen in clinical practice (1).

### 2.2 Why passive scaffolds fail

Underlying the outcome data summarized in Section 1.2 is a crucial biological reality: all currently approved NGCs, and even processed allografts, are fundamentally passive scaffolds. They can guide axons mechanically, but they cannot actively modulate the inhibitory biochemical and cellular microenvironment described above. Passive NGCs cannot degrade CSPGs, neutralize myelin-associated inhibitors, quench oxidative stress, or reprogram the local immune-cell environment from a pro-inflammatory to a pro-regenerative phenotype.

This recognition has galvanized the field toward what Rochkind has termed “neurogels” (5): bioactive hydrogels designed not only to bridge nerve gaps structurally but to actively modulate the biochemical and cellular microenvironment, suppressing scarring, providing axonal guidance signals, countering oxidative injury, and immunomodulating the post-injury inflammatory cascade. The development and optimization of such neurogels have been the focus of more than two decades of work by Rochkind and Nevo, and constitute the subject of the present review. Section 3 develops the design rationale for each component of this bioactive strategy.

## 3 Design rationale for a bioactive neurogel

### 3.1 The neurogel concept: from passive scaffold to active modulator

The peripheral and central nervous systems are embedded within an extracellular matrix that performs far more than a structural support role: it provides contact-mediated guidance cues, reservoir functions for neurotrophic factors, physical scaffolding for migrating Schwann cells, and biochemical signals that regulate neuronal survival, axon pathfinding, differentiation, and myelination (34, 35). In fetal and neonatal tissues, this ECM is particularly rich in hyaluronic acid and laminin glycoproteins, conditions associated with rapid, scar-free wound healing and robust neural development (36). The design logic underpinning GRG and AGRG was to recapitulate this permissive fetal-like matrix within the lumen of an NGC or at a spinal cord injury site, providing regenerating axons with the molecular guidance cues, hydration, antioxidant protection, and, in the AGRG formulation, anti-inhibitory modulation necessary to traverse what would otherwise be an unbridgeable gap.

A hydrogel, a three-dimensional, water-swollen polymer network, is uniquely suited to this purpose (37). Hydrogels can be formulated to match the soft viscoelastic properties of neural tissue, delivered in liquid form to fill irregularly shaped defects, and engineered to release bioactive agents in a sustained, controlled manner (37, 38). Unlike solid scaffolds, hydrogels do not create fibrotic capsules and can be resorbed as regenerating tissue replaces them.

### 3.2 Hyaluronic acid backbone

Hyaluronic acid (HA), also known as hyaluronan, is a naturally occurring, non-sulfated glycosaminoglycan polymer of alternating D-glucuronic acid and N-acetyl-D-glucosamine disaccharide units, present in virtually all mammalian connective tissues and particularly enriched in the CNS ECM (36). High-molecular-weight HA (>1.5 MDa, as used in GRG and AGRG) contributes several properties directly relevant to nerve regeneration. First, HA's extraordinary capacity for water retention, a single molecule binds up to 1,000 times its weight in water, sustains the hydrated microenvironment that prevents drying of regenerating axons during the critical early post-repair period (36, 39). Second, HA functions as an antioxidant at the aqueous phase, scavenging reactive oxygen species generated by post-injury ischemia-reperfusion and inflammatory cell activity, complementing the lipid-phase antioxidant protection provided by α-tocopherol (39). Third, HA serves as a versatile slow-release vehicle for the other active components, the laminin peptide, DL-α-tocopherol, and glatiramer acetate, enabling sustained local delivery at the injury site (36, 38). Fourth, fetal tissues are characterized by exceptionally high HA content, a feature correlated with the rapid, scarless wound healing that occurs *in utero*, in striking contrast to the fibrotic scarring of adult tissues (36, 40). By recreating this high-HA milieu, GRG and AGRG aim to recapitulate the permissive regenerative conditions of the fetal nervous system within the adult injury environment.

Beyond its biochemical roles, the physical viscoelastic behavior of the 0.4% (w/v) high-molecular-weight HA hydrogel used in GRG and AGRG is critical to its function as a nerve repair matrix. Neural tissue is among the softest tissues in the body, with an elastic (Young's) modulus in the range of 0.1–1 kPa for peripheral nerves and approximately 0.02–0.1 kPa for spinal cord parenchyma (37, 38). A scaffold that is mechanically stiffer than the surrounding tissue creates stress concentration at the scaffold-tissue interface, promotes fibroblast activation, and triggers a foreign-body reaction characterized by capsule formation and fibrosis. Conversely, a scaffold too soft to maintain structural integrity may collapse within the NGC lumen, re-creating the void that promotes fibrin scaffold failure in hollow NGCs. At 4 mg/mL HA (>1.5 MDa), the AGRG hydrogel exhibits viscoelastic behavior with storage modulus (G′) in the range of 1–10 Pa and loss modulus (G″) lower than G′ across physiological shear rates, behavior consistent with a weak physical gel that flows under surgical manipulation (allowing injection into NGCs) yet recovers structural integrity *in situ* (37). This shear-thinning, self-healing rheological profile is mechanically well matched to peripheral nerve tissue, and high-MW HA chains also entangle sufficiently to resist rapid diffusion of AGRG's other components out of the NGC lumen, enabling sustained local delivery at the repair site (38).

### 3.3 Biomimetic laminin peptide (LDP-916)

Laminin is one of the principal glycoproteins of the basement membrane, and its role in neural development and regeneration is well established. Laminin promotes neurite extension, Schwann cell migration, neuronal survival, and axonal differentiation through interactions with cell-surface integrins and other receptors (35, 41). However, native laminin protein is a large, complex, heterotrimeric glycoprotein (molecular weight ∼900 kDa) that poses formidable practical challenges for pharmaceutical development: instability in solution, batch variability, immunogenicity risk, and manufacturing complexity.

A significant conceptual advance in the GRG formulation was the replacement of native laminin with a synthetic 16-amino-acid peptide (LDP-916) that condenses the biologically active sequences of laminin into a small, chemically defined, manufacturable molecule bearing two motifs of the laminin protein: IKVAV and YIGSR (35, 41, 42). IKVAV supports neuronal adhesion, neurite outgrowth, and differentiation, while YIGSR mediates cell attachment and migration and has been shown to promote axonal guidance (41, 42). The use of a synthetic peptide allows precise dosimetry, good manufacturing practice (GMP) with batch-to-batch consistency, and regulatory simplicity compared to a recombinant or extracted protein. At a final concentration of 10 µg/mL, the peptide provides the critical biological signals for neurite elongation, Schwann cell guidance, and neuronal survival without the immunogenic risks associated with foreign protein administration (1).

### 3.4 Antioxidant strategy

Oxidative stress from superoxide radicals and downstream reactive oxygen species contributes substantially to secondary nerve injury, axonal membrane damage, and Schwann cell dysfunction at PNI and SCI sites (39, 43). The initial GRG formulation incorporated recombinant human superoxide dismutase 1 (SOD1) as an antioxidant, reflecting this evidence base. SOD1 catalyzes the dismutation of superoxide anion to hydrogen peroxide, protecting the injury microenvironment from oxidative damage and working synergistically with HA's aqueous-phase antioxidant activity (39).

However, recombinant SOD1 presents a series of regulatory and manufacturing challenges that are significant obstacles to clinical translation: thermolability at room temperature, short shelf life without refrigeration, potential immunogenicity as a recombinant protein, batch-to-batch variability, and high manufacturing cost. For the AGRG reformulation, SOD1 was replaced by DL-α-tocopherol (vitamin E) at 3 mM. This decision was driven primarily by considerations of stability, manufacturability, and regulatory feasibility rather than by evidence that SOD1 was biologically inferior, both molecules are validated antioxidants, but they act through different mechanisms. DL-α-tocopherol is a small-molecule, lipid-soluble antioxidant that primarily protects cell membranes and lipid-rich myelin sheaths from lipid peroxidation, a distinct and complementary mechanism to HA's aqueous-phase antioxidant action and to SOD1's enzymatic dismutation of superoxide (44, 45). It is also heat-stable, has a well-characterized safety profile, is commercially available in GMP-grade form, and is straightforward to manufacture reproducibly. The final DMSO concentration of approximately 0.6% resulting from the tocopherol stock solution preparation falls well below concentrations associated with cytotoxicity (1). Together, HA and DL-α-tocopherol provide complementary aqueous-phase and lipid-phase antioxidant coverage across the hydrophilic and lipophilic compartments of the regenerative microenvironment. Section 5.3 addresses directly how this substitution, made concurrently with the introduction of glatiramer acetate, complicates attribution of AGRG's efficacy gain over GRG to any single component.

### 3.5 Antigliotic modulation

Antioxidant protection and laminin-mimetic guidance address two of the principal barriers to regeneration, but neither directly counters the CSPG deposition and myelin-associated inhibition that dominate the chronic glial scar (Section 2.1). This recognition motivated the addition of a third functional class of component, an antigliotic agent capable of modulating the inhibitory post-injury immune and glial environment, culminating in the transition from GRG to AGRG. The scientific process by which candidate antigliotic agents were identified, and the rationale for the specific agent ultimately selected (glatiramer acetate), are described in Section 4.

## 4 Formulation evolution: from HA hydrogels to AGRG

### 4.1 Early hydrogel systems (1990s–2000s)

The earliest precursor of GRG emerged from research in the laboratories of Rochkind and Nevo at Tel Aviv University in the early 1990s. Building on Nevo's foundational work in proteoglycan biochemistry and ECM biology (46, 47), the group developed a first-generation hydrogel carrier in which HA served as the primary matrix, loaded with embryonal spinal cord nerve cells cultured on biodegradable microcarriers. The rationale was grounded in developmental neurobiology: embryonal neural cells retain the growth-promoting and myelination-supporting properties of the fetal nervous system, and their transplantation into an adult SCI site, mediated through a supportive HA carrier, might transfer those properties to the otherwise inhibitory adult CNS environment. In a foundational study, this composite implant, combined with low-power laser irradiation, demonstrated evidence of axonal continuity across the injury site and partial functional recovery in a rat model of traumatic paraplegia (48), providing proof of concept that a cell-loaded HA hydrogel could serve as a permissive bridge in a spinal cord lesion. This work received first-prize recognition at the 1997 World Federation of Neurosurgical Societies (WFNS) Congress.

The second generation of the hydrogel system, developed in the early 2000s, replaced the embryonal cell payload with biochemical guidance cues, reflecting a strategic shift toward a fully cell-free, biomolecular approach that would ultimately be more tractable for clinical translation and regulatory approval. This formulation comprised cross-linked high-molecular-weight HA as the backbone, supplemented with human laminin, additional ECM components, and growth factors. A polymer NGC filled with this second-generation hydrogel was used for reconnection of completely transected peripheral nerve in a rat model, yielding evidence of axonal sprouting through the gap inside the NGC (49), and further work extended the evidence base in spinal cord models, including a tissue-engineered composite implant for treatment of traumatic paraplegia in rats reported in the *European Spine Journal* (50). These studies established the principle of combining HA with laminin-family proteins and growth factors in a single injectable matrix, and generated the foundational intellectual property upon which subsequent GRG formulations were built.

### 4.2 The new hydrogel program (2011–2015)

Between 2011 and 2015, a refined “New Hydrogel” formulation was developed as part of an EU FP7-funded collaborative program, with the specific objective of advancing HA-based hydrogels toward clinical application in peripheral nerve reconstruction. This formulation retained high-molecular-weight HA and human laminin as core constituents, with the addition of antioxidant components to address the role of oxidative stress at the injury site. The project brought together academic and industrial partners and generated important formulation data on gel rheology, biocompatibility, and *in vitro* and *in vivo* nerve regeneration. Results from this program were reported by Meyer and colleagues in a comprehensive study of peripheral nerve regeneration through hydrogel-enriched chitosan NGCs, which demonstrated the capacity of hydrogel-filled NGC to support Schwann cell survival and axonal guidance in an established rat model (51). This EU FP7 program served as a critical bridge between the academic proof-of-concept experiments of the 1990s and early 2000s and the definitive GRG formulation that followed.

### 4.3 GRG: HA + LDP-916 + SOD1 (2011–2013)

This phase of the GRG project emerged from a collaborative research program conducted between 2011 and 2013 with support from a research grant provided by Teva Pharmaceutical Industries. The key innovation was the replacement of native human laminin with the laminin-derived chimeric 16-amino-acid peptide (LDP-916) described in Section 3.3, incorporating the IKVAV and YIGSR bioactive motifs. Recombinant SOD1 was included as the antioxidant component (Section 3.4). Together with hyaluronic acid, these components formed a ternary system (HA + LDP-916 + SOD1) that yielded a highly viscous, transparent, flexible, and injectable gel that could be readily injected into NGCs and conform to a wide range of NGC configurations and defect geometries (39). The associated intellectual property was protected by patent PCT WO 2009/022339.

The critical preclinical validation of GRG was reported by Rochkind and Nevo in *BioMed Research International* in 2014 (39). This double-blind, randomized study enrolled 32 Wistar rats assigned to four equal groups (*n* = 8 per group): (1) autologous peripheral nerve graft (ANG, gold standard); (2) bridging with a standard hollow collagen NeuraGen® Nerve Guide; (3) bridging with a NeuraGen® Nerve Guide filled with HA vehicle only; and (4) bridging with a NeuraGen® Nerve Guide filled with GRG. The sciatic nerve was completely transected and a 10 mm nerve segment was excised, creating a reconstructed gap of 15 mm within a 17 mm NGC. This gap length exceeds the regenerative capacity of hollow NGC in the rat model, in which spontaneous regeneration is possible only for gaps of up to 7 mm. All animals were followed for 90 days, after which the operated sciatic nerve was harvested for histological analysis at three sites, using a blinded six-point histological scale (0 = scar tissue, no axons; 5 = similar to proximal nerve).

The results provided unequivocal support for GRG's regenerative capacity. In the hollow NGC group, no axonal growth was observed within the NGC. In the GRG group, growth of myelinated axons was evident within the NGC, and axonal sprouting continued through the NGC to the distal nerve segment (*p* < 0.001 vs. hollow NGC). GRG's myelinated axon growth to the distal segment was significantly superior to the HA-only group (*p* < 0.014), demonstrating that the bioactive components beyond HA, specifically LDP-916 and SOD1, were essential contributors rather than HA alone. Critically, no significant histological difference was found between the GRG group and the ANG group (39): GRG enabled axonal regeneration approaching the gold-standard level across a 15 mm critical gap that was otherwise unbridgeable with existing NGC technology. The GRG group achieved mean scores in the range of 3–4 within the NGC and at the distal site, while the ANG group achieved scores of 4–5 at both locations; the HA-only group achieved scores of 2–3, and the hollow NGC group scored 0–1 within the NGC with no axonal presence distally. The absence of a statistically significant difference between GRG and ANG on this scale, despite GRG being a fully cell-free, injectable biomolecular gel, validated the design principle that a biochemically active hydrogel can functionally approximate the biological richness of the autograft without its structural complexity or donor-site morbidity. A subsequent functional study over six months supported this conclusion (1).

### 4.4 The pivot to AGRG: rationale for glatiramer acetate and DL-α-tocopherol

#### 4.4.1 The *in vitro* antigliotic candidate-selection screen

Following the success of GRG, the group turned to the specific problem of glial scar formation described in Section 2.1, and undertook an internal candidate-selection program to identify agents capable of suppressing CSPG accumulation by activated astrocytes. This program used a quantitative *in vitro* assay to rank candidate antigliotic agents before considering their incorporation into the AGRG matrix. The assay exploited the capacity of activated astrocytes to accumulate ^35^S-labeled glycosaminoglycans (^35^S-GAGs), the biochemical correlate of glial scar formation, using the human astrocytoma cell line U-87 MG activated by theophylline (0.25 mM) and N6,2′-O-dibutyryladenosine 3′,5′-cyclic monophosphate (Bt2-cAMP, 1 mM), pharmacological mimics of the astrocyte-activating signals associated with CNS injury. Eight candidate antigliotic agents were evaluated in triplicate, with GAG accumulation measured as radioactivity counts per minute normalized to the theophylline-activated control (100%). This internal screen was conducted as a candidate-selection tool to prioritize agents for the AGRG formulation program; it has not been separately published in full, and its findings are summarized here directionally, in the context of the formulation decisions they informed, rather than as an independently verified dataset in their own right.

**Figure 1:**
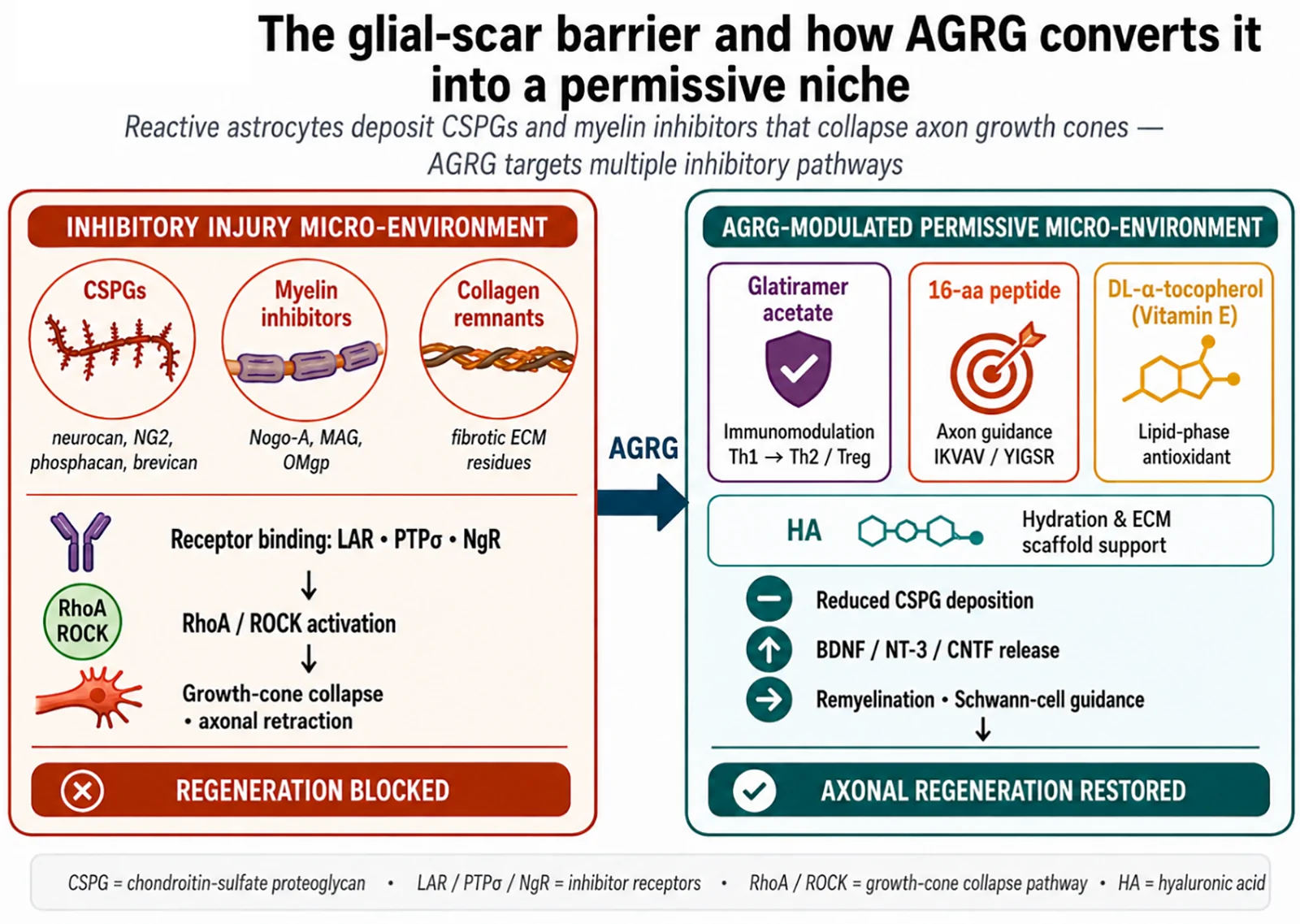
Reversal of glial-scar-mediated inhibition of axonal regeneration by AGRG therapy. Inhibitory cues within the injury microenvironment – CSPGs, myelin-associated inhibitors, and collagen remnants – block axonal regrowth (left panel). AGRG components – glatiramer acetate, a 16-amino-acid peptide, vitamin E, and hyaluronic acid – convert this barrier into a regeneration-permissive environment by enhancing hydration, limiting CSPG deposition, and stimulating growth-factor release (right panel).

The rank-ordered results from this internal screen provided direction for the AGRG formulation. When normalized to the theophylline control (which increased ^35^S-GAG accumulation more than six-fold above the unstimulated baseline), the most potent antigliotic candidates identified were: chondroitinase A, B & C, which reduced GAG accumulation to 5.5% of theophylline control (approximately 20-fold potency relative to theophylline); anti-human NogoA, which reduced GAG accumulation to 15.3% of control (6.5-fold potency); anti-TGFβ 1,2&3, which reduced accumulation to 19.8% of control; mitomycin C, to 20.2% of control; and recombinant human angiotensin-converting enzyme (ACE), to 22.9% of control (Figure 1). Of the eight agents tested, five demonstrated significant antigliotic activity. This screen was used as an internal, hypothesis-generating step to guide the subsequent formulation program described in Section 4.4.2 and 4.5; it was not, by itself, sufficient evidence to select a final clinical candidate, because the top-ranked biologics carried translational limitations addressed below.

**Figure 2:**
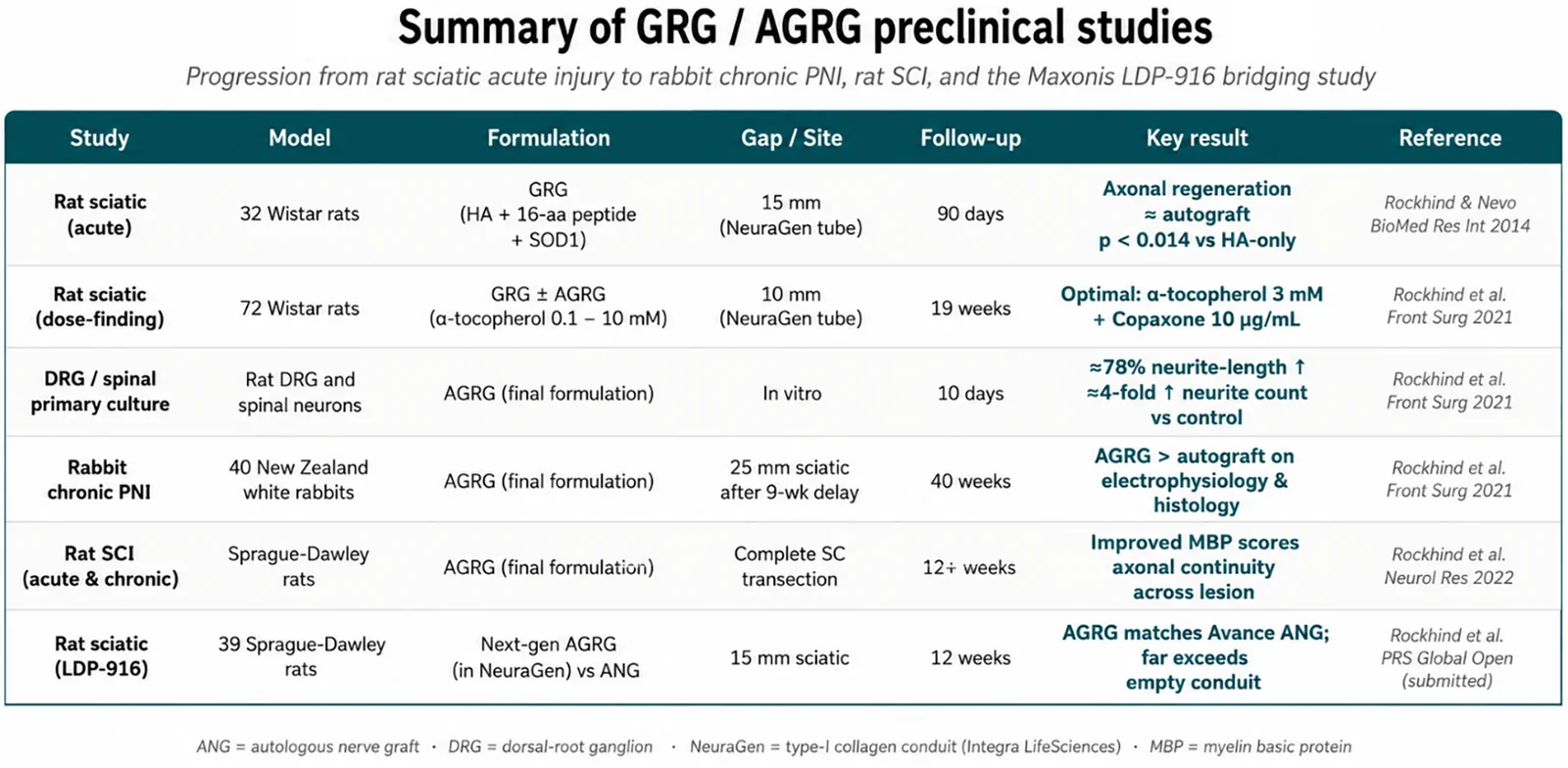
Summary of GRG/AGRG preclinical studies. Species, model, gap length, treatment groups, and principal electrophysiological/histochemical outcomes across the rat sciatic 15 mm acute-gap, rat 10 mm dose-finding, rabbit chronic 25 mm-gap, rat acute and chronic complete SCI, and the rat 15 mm critical-gap studies.

**Figure 3:**
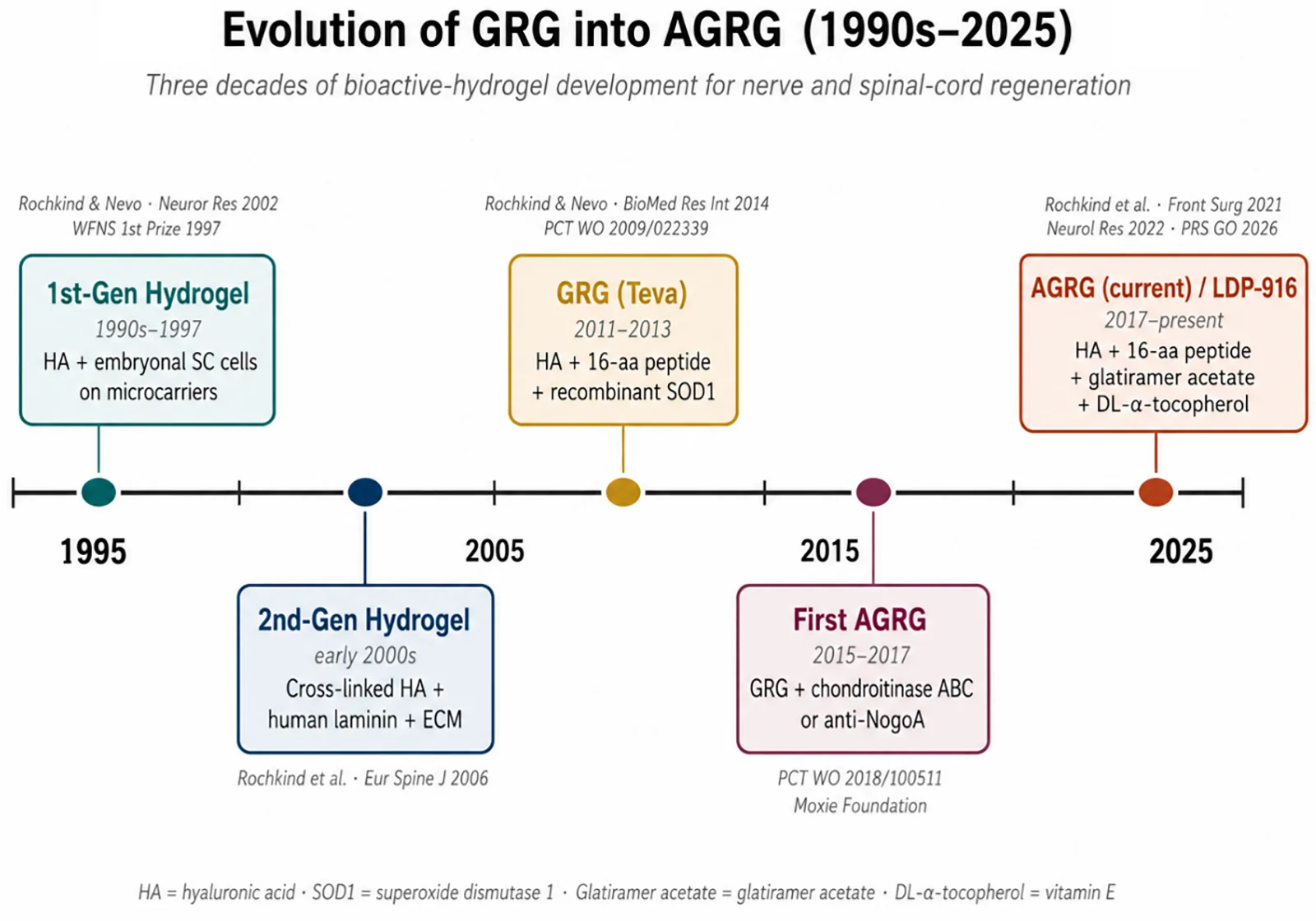
Timeline of GRG → AGRG development, 1990s–2025. Sequence of formulations, funding milestones, and key publications spanning the first-generation HA + embryonal cell hydrogel through to AGRG/LDP-916.

#### 4.4.2 Fifth-generation AGRG with biologic antigliotics (2015–2017)

Armed with the internal screening data, the group developed the next generation of the GRG formulation, initially termed AGRG (Antigliotic Guiding Regenerative Gel), between 2015 and 2017 with support from a grant provided by the Moxie Foundation. Two antigliotic agents were initially selected based on their demonstrated *in vitro* efficacy in the candidate-selection screen: chondroitinase ABC, an enzyme that degrades the GAG side chains of CSPGs, thereby dismantling a principal inhibitory component of the glial scar (33, 52); and an anti-NogoA antibody, which neutralizes the myelin-associated neurite growth inhibitor Nogo-A. These early AGRG variants were evaluated in preclinical SCI models and provided proof of concept that enriching GRG with targeted antigliotic agents could attenuate glial scar formation and facilitate axonal regeneration across the lesion site. The resulting intellectual property was protected under patent PCT WO 2018/100511.

The rationale for incorporating chondroitinase ABC was strongly supported by extensive prior research demonstrating its ability to promote functional recovery following SCI (52–54), enhance plasticity of spinal sensory pathways (55), stimulate sprouting of both intact and injured spinal tracts (56), and facilitate axonal regeneration through Schwann cell-seeded guidance channels (57). Likewise, neutralization of Nogo-A using the IN-1 antibody and related constructs has been shown to promote axonal regeneration and functional recovery in experimental SCI models (24, 28). Collectively, these findings provided strong scientific justification for the development of AGRG as a multifunctional regenerative platform capable of addressing both the permissive ECM requirements for regeneration and the inhibitory mechanisms associated with glial scar formation.

#### 4.4.3 Rationale for replacing chondroitinase ABC and anti-NogoA with glatiramer acetate

Despite their demonstrated efficacy in preclinical studies, both chondroitinase ABC and anti-NogoA antibody present significant translational challenges that limit their practical clinical application, and it is the resolution of this translational impasse, not a change in the underlying scientific target, that led to the current AGRG formulation.

Chondroitinase ABC is derived from *Proteus vulgaris*, a bacterial origin that carries inherent immunogenicity risk in human subjects and necessitates extensive bioburden testing and depyrogenation for pharmaceutical manufacture. The enzyme is thermolabile, with rapid activity loss at 37 °C (body temperature), creating formidable challenges for maintaining consistent biological activity during *in vivo* delivery and storage (58). There is currently no FDA-approved human formulation of chondroitinase ABC; clinical application would require a full biological license application under a *de novo* regulatory pathway. These considerations do not negate chondroitinase ABC's biological efficacy, but they create a practical barrier that has significantly delayed its clinical application despite decades of preclinical proof of concept.

Anti-NogoA humanized monoclonal antibody requires intrathecal or intraparenchymal delivery to achieve adequate CNS concentrations, given the blood-brain barrier's impermeability to large antibody molecules; systemic delivery does not achieve therapeutically relevant CNS concentrations. Intrathecal delivery involves a separate invasive procedure with associated infection and hemorrhage risk, and the therapeutic window for anti-NogoA, optimal within the first weeks of injury before glial scar consolidation, imposes additional logistical demands. Manufacturing humanized monoclonal antibodies at clinical-grade quality represents a major development investment (59).

Glatiramer acetate (GA), by contrast, is a well-documented and globally approved active pharmaceutical ingredient used in the treatment of multiple sclerosis, marketed as Copaxone (Teva Pharmaceutical Industries). Its established regulatory status, large-scale manufacturing infrastructure, and extensive clinical safety record made it an attractive candidate to resolve the translational impasse presented by the biologic antigliotics. GA had appeared among the eight candidates in the original internal screen (Section 4.4.1) without ranking among the most potent GAG-reducing agents in that assay; its selection for the final AGRG formulation was driven principally by its translational advantages rather than by superior potency in the screen, and this distinction is addressed explicitly in Section 5.3 and in the response to Reviewer 2 accompanying this revision.

GA is a random copolymer of four amino acids, L-glutamic acid, L-lysine, L-alanine, and L-tyrosine, in molar ratios of approximately 1.4:3.4:4.2:1.0, with a mean molecular weight of approximately 5–9 kDa. It has been FDA-approved for the treatment of relapsing-remitting multiple sclerosis since 1996 (60), accumulating a human safety record across millions of patient-years of subcutaneous daily injection.

The immunomodulatory mechanisms of GA are well characterized in the MS literature (61–63). It acts as a partial antigen by competing with myelin basic protein (MBP) for binding to MHC class II molecules on antigen-presenting cells, thereby driving a shift in the T-helper cell balance from the pro-inflammatory Th1 phenotype toward the anti-inflammatory Th2 and regulatory T-cell (Treg) phenotype (61, 62). This immunological shift has broad consequences for the post-injury neuroinflammatory environment: Th2 cells and Tregs suppress the macrophage and microglial pro-inflammatory activation that amplifies CSPG deposition and secondary axonal injury. GA-reactive T cells produce and release brain-derived neurotrophic factor (BDNF), ciliary neurotrophic factor (CNTF), and neurotrophin-3 (NT-3) at the site of CNS pathology, providing a direct neurotrophic stimulus that neither chondroitinase ABC nor anti-NogoA delivers (63, 64). GA has further been shown to promote remyelination through support of oligodendrocyte progenitor cell survival and differentiation, a potential advantage for SCI applications where remyelination is as important as axonal regrowth (65). Documented neuroprotective effects in optic neuritis, glaucoma, and experimental autoimmune encephalomyelitis (EAE) models provide additional evidence of GA's capacity to protect neural tissue through mechanisms that extend beyond its role as an MS immunomodulator (66, 67).

##### 4.4.3.1 Local vs. systemic mechanism

Within the AGRG matrix, GA is delivered locally rather than systemically, and its mechanism of action in this context merits explicit mechanistic discussion because it differs from the classical MS paradigm in which GA is injected subcutaneously and its effects are mediated substantially by a systemically primed T-cell repertoire. Two complementary, non-mutually-exclusive modes of action are biologically plausible for locally delivered GA within an NGC lumen or spinal cord lesion. First, at the local level, high concentrations of GA released from the HA hydrogel can act directly on antigen-presenting cells, infiltrating monocytes, macrophages, and resident microglia, within the injury site, engaging MHC class II independent of any requirement for a pre-existing systemic pool of GA-specific T cells; this local action drives macrophage/microglial polarization toward an anti-inflammatory M2 phenotype and secretion of IL-10 and neurotrophic factors including BDNF, consistent with extensive prior work on GA's local immunomodulatory mechanism in the MS and experimental autoimmune encephalomyelitis literature (61–64). Second, at the systemic level, GA released from the implant can drain via lymphatics to regional lymph nodes, where it can prime a GA-reactive T-helper-2 (Th2) population even without prior subcutaneous immunization; these peripherally generated Th2 cells can subsequently traffic to the CNS or PNS injury site along chemokine gradients associated with “protective autoimmunity,” a concept developed extensively in the work of Schwartz, Kipnis, Aharoni, and colleagues in the context of GA and CNS injury (62, 66, 67). The relative contribution of these two modes within the AGRG/NGC context has not been directly dissected by immunocellular studies to date; the current preclinical evidence for AGRG rests on functional (electrophysiological, histological, behavioral) endpoints rather than on direct immunophenotyping of the injury site, and we identify this as an open mechanistic question in Section 7.2 and in our response to Reviewer 2's Comment 4.

In summary, GA addresses the inhibitory post-injury microenvironment through a fundamentally different and complementary mechanism compared to chondroitinase ABC or anti-NogoA: rather than enzymatically degrading CSPGs or neutralizing Nogo-A protein, it reprograms the local and regional immune-cell environment toward a phenotype that supports regeneration and suppresses scar formation, while bringing FDA-approved safety credentials, GMP availability, established manufacturing infrastructure, and water solubility in the hydrogel vehicle (1, 5, 60, 61).

##### 4.4.3.2 Concurrent replacement of SOD1 with DL-α-tocopherol

The transition from GRG to AGRG also involved replacement of recombinant SOD1 with DL-α-tocopherol as the antioxidant component, for the manufacturability and stability reasons discussed in Section 3.4. Because this antioxidant substitution occurred concurrently with the introduction of glatiramer acetate, the two changes are confounded in any comparison of GRG vs. AGRG efficacy; Section 5.3 addresses this confounding directly, as raised by peer review of this manuscript.

### 4.5 Final AGRG composition

The current AGRG formulation that has been evaluated in *in vitro*, rat, and rabbit models comprises four precisely specified components (1, 68):

- High-molecular-weight hyaluronic acid: structural hydrogel backbone.
- 16-amino-acid laminin-derived peptide (LDP-916): promotes neurite outgrowth, Schwann cell migration, neuronal survival.
- DL-α-tocopherol: lipid-phase antioxidant.
- Glatiramer acetate: antigliotic immunomodulator, neurotrophic factor induction.

## 5 Preclinical evidence

### 5.1 *In vitro* neurite outgrowth

The initial *in vitro* characterization of AGRG was performed on two cell systems: primary dorsal root ganglion (DRG) neuron cultures from embryonic mice, and primary spinal cord cultures from one-month-old Sprague-Dawley rats. In primary spinal cord cultures, the AGRG formulation demonstrated a significant enhancement of neuronal outgrowth, with mean neurite length per cell increasing by approximately 78% relative to untreated control cultures; the total number of neurites increased by approximately four-fold over the same comparison (1). AGRG also outperformed the original GRG formulation across key neurite outgrowth parameters. Because this *in vitro* comparison isolates the AGRG-vs.-GRG difference to the combined effect of the antigliotic (chondroitinase ABC/anti-NogoA-informed candidate selection culminating in glatiramer acetate) and antioxidant (tocopherol-for-SOD1) substitutions in a cell-free system without an immune compartment, it provides evidence that the antigliotic and/or antioxidant changes, rather than an immune-cell-dependent mechanism, can account for at least part of AGRG's incremental benefit over GRG, a point directly relevant to the confounding discussed in Section 5.3. Collectively, these *in vitro* findings established AGRG as the most effective formulation tested for promoting neuronal outgrowth, and provided the rationale for advancing to *in vivo* dose-finding and efficacy studies.

### 5.2 Dose-finding in rat sciatic gap

Before proceeding to a rabbit efficacy study, a systematic dose-optimization study was performed in a rat acute PNI model (1). Seventy-two male Wistar rats (250–300 g) underwent creation of a 10 mm sciatic nerve gap and reconstruction with a collagen NeuraGen® Nerve Guide. Animals were allocated to groups receiving GRG with DL-α-tocopherol at four concentrations (0.13, 0.43, 1.29, and 4.32 mg/mL), with and without glatiramer acetate at 10 µg/mL. Electrophysiological and histochemical assessments were performed at 19 weeks. This study identified 1.29 mg/mL DL-α-tocopherol as the optimal concentration, balancing antioxidant efficacy with absence of cytotoxicity at higher concentrations. The combination of 1.29 mg/mL DL-α-tocopherol with 10 µg/mL glatiramer acetate provided the most favorable neuronal outgrowth profile and was selected as the definitive AGRG formulation for subsequent *in vivo* efficacy studies (1). This systematic optimization approach ensures that the final formulation delivers each component at its biologically effective concentration without pharmacological interference between components.

### 5.3 Chronic rabbit PNI model: 25 mm critical gap

The principal *in vivo* efficacy study for AGRG was conducted in a rabbit model of chronic peripheral nerve injury with massive loss defect, a model specifically designed to replicate the clinical scenario of delayed reconstruction in a patient with a large, chronically denervated nerve gap (1). This model is more clinically relevant than acute rat sciatic nerve models for two reasons: first, the 25 mm gap length exceeds the regenerative capacity of any currently available NGC in the rabbit; and second, the nine-week delay between complete transection and reconstruction creates the denervated, fibrotic, CSPG-enriched environment characteristic of real-world clinical presentations.

#### 5.3.1 Study design

Forty New Zealand white rabbits underwent complete transection of the tibial portion of the sciatic nerve at the first surgery. Nine weeks later (second surgery, mimicking delayed/chronic PNI), a 25 mm gap was created by nerve segment resection and reconstructed by one of four methods: ANG, hollow collagen NeuraGen® Nerve Guide, NeuraGen® Nerve Guide filled with GRG, or NeuraGen® Nerve Guide filled with AGRG. Animals were followed for 40 weeks after the first surgery, with electrophysiological (compound muscle action potential, CMAP) and histochemical assessments performed at sequential timepoints (1).

#### 5.3.2 Electrophysiological results

AGRG treatment provided electrophysiological recovery superior to or comparable with the autologous nerve graft group, and substantially superior to hollow NGC and GRG groups, across the critical 25 mm gap. The beneficial effect of AGRG vs. autologous nerve graft was statistically significant at multiple follow-up timepoints (1).

#### 5.3.3 Histochemical results

At 31 weeks following the second (delayed reconstruction) surgery, histochemical analysis demonstrated significant regeneration in the AGRG group compared to the hollow NGC group, with axonal continuity observed across the reconstruction site. The histochemical appearance in AGRG-treated animals approached that of a healthy, near-normal nerve at the 40-week endpoint, exceeding what was observed in the GRG and hollow NGC groups and comparable to the autologous nerve graft group (1).

#### 5.3.4 Addressing the SOD1/tocopherol/glatiramer-acetate confounding

The superior performance of AGRG compared to GRG in this study reflects two simultaneous formulation changes, the replacement of SOD1 by DL-α-tocopherol and the introduction of glatiramer acetate as the antigliotic component (Section 4.4), and this study design cannot, by itself, unambiguously assign the observed efficacy gain to either change individually or apportion their relative contributions. This is an important limitation that we acknowledge explicitly. Several lines of indirect evidence, discussed in detail in our response to Reviewer 2, are nonetheless informative: the *in vitro* antigliotic screen (Section 4.4.1) demonstrates glatiramer acetate's capacity to influence glial GAG accumulation through a mechanism that is independent of antioxidant activity; the extensive MS literature establishes that glatiramer acetate exerts immunomodulatory and neuroprotective effects *in vivo* without any accompanying antioxidant reformulation (61–64, 66, 67); and both SOD1 and DL-α-tocopherol are independently validated antioxidants with distinct but overlapping mechanisms, making it mechanistically plausible that the antioxidant substitution alone contributed comparatively little to the observed efficacy difference, while the introduction of an antigliotic, immunomodulatory agent represents the more probable single largest contributor. However, we regard this as a plausible inference rather than a demonstrated conclusion. Because SOD1 and the earlier GRG formulation have been retired from further development for the manufacturability and stability reasons discussed in Section 3.4 and Section 4.3, a retrospective dissection of the GRG-to-AGRG efficacy gain is no longer part of the forward research plan. Instead, the forward-looking priority experiment, described in Section 7.2, will isolate the contribution of DL-α-tocopherol within the current AGRG platform by comparing AGRG with and without DL-α-tocopherol against autograft, using the established endpoints of the rat sciatic critical-gap and rabbit chronic PNI models.

### 5.4 Acute and chronic rat SCI

The applicability of AGRG beyond peripheral nerve injury to the more challenging terrain of spinal cord injury was investigated by Rochkind, Almog, and Nevo, with results published in *Neurological Research* in 2022 (2). This study evaluated AGRG in rat models of both acute and chronic complete SCI at the Th7-Th8 thoracic level.

#### 5.4.1 Acute SCI

In the acute SCI model, a 2 mm segment of spinal cord was excised at Th7-Th8, creating a complete transection gap. AGRG was injected directly into the gap immediately after creation, with untreated animals serving as controls. At 24 weeks, 75% of AGRG-treated animals demonstrated somatosensory evoked potential (SEP) signal recovery, electrophysiological evidence of signal conduction across the lesion site. Immunohistochemical analysis revealed significantly greater expression of myelin basic protein (MBP) in the injured area in the AGRG group compared to untreated controls (*p* < 0.1), indicating remyelination of regenerating axons in the AGRG-treated lesion zone (2).

#### 5.4.2 Chronic SCI

In the chronic SCI model, a 1 mm spinal cord segment was removed at Th7-Th8. One month later, the injured area, now characterized by established glial scar, CSPG accumulation, and cavitation, was surgically debrided to remove connective and scar tissue, creating a 2–3 mm gap, into which AGRG was then injected. At 24 weeks after the second surgery, significantly greater MBP expression was observed in the AGRG group compared to untreated controls (*p* < 0.01), indicating myelinated axon growth across the chronic SCI lesion in AGRG-treated animals (2). This result is particularly noteworthy because the chronic SCI model represents the most clinically relevant scenario for most SCI patients.

These results demonstrate that AGRG's antigliotic components provide meaningful biological activity in the CNS environment, reducing glial scar inhibition sufficiently to permit myelinated axon regeneration across a lesion that is otherwise irreparable in the adult rat.

### 5.5 Rat 15 mm sciatic gap (recent work)

A further evaluation of the AGRG platform, conducted under the commercial development program of Maxonis Ltd., is a prospective, randomized, blinded study of 39 Sprague-Dawley rats comparing AGRG (LDP-916) against ANG and hollow collagen NeuraGen® Nerve Guide in a 15 mm sciatic nerve critical-gap model, with a comprehensive outcome battery including von Frey sensory testing, CMAP electrophysiology, MBP expression, synaptophysin neuromuscular junction (NMJ) staining, and gastrocnemius muscle atrophy scoring. This study has been submitted for independent peer review and is not yet published; consistent with standard scientific practice for a review article, we describe its design and general direction of findings here without presenting unreviewed numerical results as an established evidentiary basis, and we do not rely on this study for any of the conclusions of this review. The study allocated 39 female Sprague-Dawley rats to four groups: intact controls, ANG, hollow NeuraGen® Nerve Guide, and NeuraGen® Nerve Guide filled with AGRG, with assessment at 1, 2.5, and 4 months across the outcome battery described above. In general terms, preliminary internal analysis suggests performance of AGRG comparable to or exceeding autograft across several of these endpoints; because this dataset has not completed independent peer review, we regard it as internally supportive but not yet a validated part of the published evidence base, and we will update the record according to the outcome of that review. This study, once published, is intended to serve as a bridging dataset supporting the translational program described in Section 7.1.

Figure 2 summarizes the preclinical studies of both formulations, and Figure 3 illustrates the evolution of GRG into AGRG.

## 6 AGRG in the broader landscape of nerve repair technologies

### 6.1 Comparative meta-analytic evidence

The most rigorous quantitative synthesis of outcomes across nerve repair modalities to date is the systematic review and meta-analysis published by Lans, Eberlin, Evans, and colleagues in *Plastic and Reconstructive Surgery* in 2023 (8). This analysis included 1,559 nerve repairs from 35 clinical studies, spanning nerve gap defects from >5 mm to 70 mm, and compared meaningful recovery (MR, defined as ≥S3/M3 MRC grade) across processed allografts, autografts, and hollow NGCs. The pooled MR rates were: allograft 87.1%; ANG 81.6%; NGC 62.2%. The NGC MR rate was statistically significantly inferior to both autograft and allograft (*p* < 0.05). Importantly, even these aggregate figures may overestimate NGC performance because the analyzed studies skew toward shorter, sensory gaps where NGCs perform best; as gap length increases toward and beyond 30 mm, NGC MR approaches 50%–60% even in sensory nerves, and falls further in motor and mixed nerves.

The Lans meta-analysis also addressed short-gap (>5 to ≤30 mm) vs. long-gap (>30 to ≤70 mm) performance. In short gaps, allografts and autografts achieved equivalent MR, while NGC MR was approximately 60% (8). In long gaps, allografts maintained meaningful recovery approaching autograft levels, while NGC performance was essentially inadequate. NGC-treated patients showed notably higher rates of persistent neuropathic pain, sensory disturbance, and symptomatic neuroma formation. Economic analysis indicated that allograft procedures did not incur higher total costs than autografts once donor-site surgery time, hospital stay, and harvest-related morbidity were factored in (8). These data establish that the current technology ceiling for hollow NGCs sits at approximately 30 mm in sensory nerves, with rapid performance decline above this threshold.

### 6.2 Taxonomy of current clinical NGCs

Currently available FDA-approved nerve repair NGCs span three main material categories, each with characteristic strengths and limitations (6, 7).

**Collagen-based NGCs**, including NeuraGen® Nerve Guide (Integra LifeSciences) and NeuraGen® 3D Nerve Guide, are constructed from Type I collagen, offering excellent biocompatibility, predictable biodegradation, and ease of surgical handling. NeuraGen hollow NGCs support early fibrin scaffold formation and Schwann cell migration but provide minimal internal architecture, limiting lumen patency in longer gaps. The next-generation NeuraGen® 3D Nerve Guide incorporates a chondroitin-6-sulfate (C6S)-infused 3D inner matrix, with preclinical data showing improved axon counts, nerve fiber density, CMAP recovery, and muscle outcomes compared to hollow NGCs (69). However, neither generation has demonstrated reliable clinical performance in defects longer than 20–30 mm or in mixed motor nerves (6).

**Synthetic polymer NGCs**, including Neurotube (polyglycolic acid, PGA), Neurolac (polycaprolactone, PCL), and SaluBridge (polyvinyl alcohol, PVA), offer mechanical strength and customizable degradation profiles (7). These NGCs can be engineered for defined porosity, stiffness, and permeability, but they often elicit foreign-body inflammatory responses and degrade unpredictably. They provide exclusively passive mechanical guidance with no neurotrophic or immunomodulatory activity.

**Processed decellularized allografts**, most notably Avance® Nerve Graft (AxoGen Inc.), preserve the intrinsic ECM architecture, including basal lamina microtubes and laminin composition, while removing immunogenic cellular components. This preserved scaffold architecture guides axonal regeneration more effectively than hollow NGCs, as documented by Kornfeld and colleagues (70). Multiple clinical studies confirm processed allograft performance comparable to autograft for defects up to 70 mm without donor-site morbidity (8). Remaining limitations include loss of viable Schwann cells during decellularization, reduced performance in motor-dominant nerves, and diminished efficacy in heavily scarred or ballistic injuries (8, 70).

Across all NGC classes, five fundamental biological limitations constrain their performance, as detailed by Stocco and colleagues (6) and de Ruiter and colleagues (7): biological inactivity (no neurotrophic, ECM-guidance, or immunomodulatory function); a sharp gap-length performance threshold around 20–30 mm; susceptibility to intraluminal fibrosis and scar formation; inability to modulate the inhibitory CSPG/Nogo-A-rich microenvironment; and poor performance in motor nerves and complex trauma. The clinical consequence is a substantial population of patients with unsatisfactory nerve repair outcomes despite technically adequate surgery. Data from Noble and colleagues, analyzing 5,777 peripheral nerve injuries in a multi-trauma population, found that complete or significant functional deficit persisted in the majority of patients with motor-dominant injuries despite surgical repair (71). A systematic review by Bergmeister and colleagues found that even with optimal microsurgical technique, recovery to functional grade M4 or above was achieved in less than 50% of motor nerve repairs, and less than 30% of patients with injury to long motor nerves achieved independent limb function (72). Asplund and colleagues reported that traumatic peripheral nerve injury in Sweden between 1998 and 2006 resulted in a 5.8-fold higher risk of long-term disability and welfare dependency compared to matched controls without nerve injury (73). These population-level outcome data underscore that the existing technology ceiling is insufficient for the scale of the clinical problem.

### 6.3 Alternative hydrogel technologies and competing regenerative strategies

AGRG is one of several bioactive strategies under active investigation for nerve repair, and a balanced assessment of its place in the field requires situating it explicitly among these alternatives, several of which pursue complementary rather than mutually exclusive mechanisms. A recent systematic review by Khavandegar and colleagues of bioengineered scaffolds for spinal cord injury, encompassing preclinical and early-phase clinical studies, provides a useful organizing framework for this broader landscape (74).

#### 6.3.1 ChABC-based approaches

As discussed in Section 4.4.2–4.4.3, chondroitinase ABC (ChABC) directly degrades the GAG side chains of CSPGs and has a strong preclinical efficacy record in SCI models (52–57). Efforts to translate ChABC clinically have focused on thermostabilized formulations and controlled-release delivery systems (including hydrogel and lentiviral-vector delivery) intended to circumvent its bacterial origin and thermolability (58). These approaches remain earlier in translational development than AGRG, but they target the identical CSPG substrate that motivated AGRG's own antigliotic candidate-selection program, and a thermostable ChABC delivery platform, if achieved, would represent a direct enzymatic complement or alternative to AGRG's immunomodulatory antigliotic strategy.

#### 6.3.2 Other bioactive hydrogels

Beyond HA-based systems, fibrin, alginate, collagen, self-assembling peptide, and decellularized extracellular matrix (dECM) hydrogels have each been investigated as nerve- and spinal-cord-repair scaffolds (74). Fibrin hydrogels offer excellent biocompatibility and can be functionalized with growth factors but degrade rapidly and provide limited intrinsic antigliotic activity. Self-assembling peptide hydrogels (e.g., RADA16-based systems) can be engineered to display bioactive epitopes analogous in concept to AGRG's LDP-916 peptide, and offer tunable mechanical properties, but most formulations to date have not incorporated an immunomodulatory antigliotic component. dECM hydrogels preserve a complex native biochemical signature and have shown promise in preclinical SCI models, but batch-to-batch compositional variability poses a translational challenge analogous to the one that motivated AGRG's move away from native laminin protein toward the synthetic LDP-916 peptide (Section 3.3). Collectively, these platforms illustrate that the field has converged on the same underlying design principle pursued by AGRG, moving beyond purely structural scaffolds toward materials with intrinsic biological activity, even where the specific bioactive strategy differs.

#### 6.3.3 Cell-based therapies

Schwann cell transplantation, mesenchymal stem cell (MSC) therapy, and induced pluripotent stem cell (iPSC)-derived neural progenitor transplantation represent a mechanistically distinct strategy that supplies living, secretory, and potentially myelinating cellular material directly to the injury site, rather than relying on a synthetic or biomolecular scaffold alone (74). These approaches can, in principle, provide a richer and more adaptive biological response than any acellular hydrogel, but face their own translational barriers, including cell sourcing and expansion, survival and engraftment after transplantation into a hostile inflammatory microenvironment, and the regulatory complexity of a cellular product. AGRG's own future direction toward cell-enriched formulations (Section 7.3) reflects direct engagement with this strategy rather than treating it as a separate competing paradigm.

#### 6.3.4 Growth factor delivery

Direct delivery of neurotrophic factors (e.g., BDNF, NT-3, glial-derived neurotrophic factor) by injection, viral vector, or controlled-release scaffold has demonstrated regenerative benefit in isolation in multiple preclinical models, but growth factor monotherapy has generally shown limited benefit as a standalone strategy in more clinically realistic chronic injury models, motivating combinatorial approaches (74). AGRG's induction of endogenous BDNF, CNTF, and NT-3 secretion via glatiramer acetate-driven immunomodulation (Section 4.4.3) can be viewed as an indirect, cell-mediated growth factor delivery strategy that avoids the pharmacokinetic and delivery challenges of exogenous protein or viral vector administration.

#### 6.3.5 Biomaterials-only vs. combinatorial strategies

The systematic review by Khavandegar and colleagues concludes that combinatorial strategies, pairing a structural/bioactive scaffold with either cellular or molecular adjuncts, generally outperform single-modality approaches in preclinical SCI models, while noting that few combinatorial strategies have yet reached early-phase clinical study (74). AGRG occupies an intermediate position on this spectrum: it is a single injectable formulation, but one that is intentionally multicomponent (structural HA backbone, biomimetic peptide, antioxidant, and immunomodulatory antigliotic agent), representing a combinatorial biological strategy delivered through a single, regulatory-tractable molecular product rather than through combination of a scaffold with a separate cellular or biologic product. This distinction has direct regulatory significance: a fully molecular combinatorial product avoids the additional manufacturing and regulatory complexity of a cell-containing combination product, at the cost of forgoing the potentially greater biological richness that living cellular components could provide.

### 6.4 How AGRG addresses key limitations

AGRG was designed specifically to overcome the limitations enumerated in Section 6.2, and each component of its formulation addresses a distinct biological deficiency of passive NGC technology. Lumen filling and structural support: by occupying the NGC lumen as a three-dimensional viscoelastic gel, AGRG eliminates the dead space that causes fibrin scaffold failure and lumen collapse in hollow NGCs (1, 39). Active biological guidance: the LDP-916 peptide provides mediated axonal guidance signals, Schwann cell migration support, and neuronal survival factors that approximate key aspects of the autograft's biological activity in a chemically defined, regulatory-tractable form (1, 41, 42). Antigliotic and immunomodulatory activity: glatiramer acetate directly addresses the inhibitory post-injury microenvironment (1, 5, 61–63), a mechanistic capability largely absent from currently approved NGCs and allografts, though shared conceptually with the ChABC-based and cell-based strategies discussed in Section 6.3. Antioxidant protection: the combination of HA (aqueous-phase) and DL-α-tocopherol (lipid-phase) provides dual-compartment antioxidant coverage (1, 39, 44, 45). Sustained hydrated microenvironment: high-MW HA maintains the water-rich, permissive milieu that characterizes the fetal wound-healing environment (1, 36).

## 7 Translational perspectives and future directions

### 7.1 Current translational status

AGRG has completed extensive preclinical evaluation across *in vitro*, rat, and rabbit PNI models and rat SCI models, summarized in Section 5. Maxonis Ltd., the biotechnology company founded to advance this technology, is currently compiling a regulatory-enabling data package, including finalized GMP manufacturing specifications, extended stability data, and a formal nonclinical safety/toxicology program, in preparation for a pre-IND meeting request to the US FDA. This represents an earlier and more clearly defined regulatory stage than a general statement of “progressing toward clinical trials”: specifically, the near-term regulatory milestone is the pre-IND interaction itself, which will define the additional nonclinical studies (if any) required before an Investigational New Drug (IND) application can be filed for a first-in-human study. Given AGRG's use of components with individually established human safety records (glatiramer acetate, DL-α-tocopherol) alongside two components without prior human exposure (LDP-916 peptide, and the specific hydrogel-delivered combination), the pre-IND interaction is expected to focus substantially on the nonclinical safety package needed to support a first-in-human PNI study.

### 7.2 Open scientific questions

Several important questions remain to be answered to optimize AGRG's clinical application and biological understanding.

#### 7.2.1 Dissecting the antioxidant and antigliotic contributions

As discussed in Section 5.3, the concurrent replacement of SOD1 with DL-α-tocopherol and the introduction of glatiramer acetate during the GRG-to-AGRG transition means that the relative contributions of these two changes to the observed efficacy gain over GRG cannot be retrospectively dissected. Because SOD1 and the earlier GRG formulation have been retired from further development for the manufacturability and stability reasons discussed in Section 3.4 and Section 4.3, no further preclinical work with SOD1-containing or GRG formulations is planned. The forward-looking priority study will instead isolate the contribution of DL-α-tocopherol within the current AGRG platform: a prospectively designed three-arm study, autograft as the reference arm, AGRG without DL-α-tocopherol (HA + LDP-916 + glatiramer acetate), and the complete AGRG formulation (HA + LDP-916 + glatiramer acetate + DL-α-tocopherol), evaluated on the established sensory, electrophysiological, histological, and muscle-preservation endpoints in the rat sciatic critical-gap and rabbit chronic PNI models. This design directly quantifies the added value of DL-α-tocopherol within the currently viable, clinically translatable AGRG formulation, and is identified here as a priority near-term study.

#### 7.2.2 Local vs. systemic glatiramer acetate mechanism

As discussed in Section 4.4.3, the relative contribution of local antigen-presenting-cell engagement vs. a lymphatically primed systemic T-cell pool to glatiramer acetate's efficacy within the AGRG/NGC context has not been directly measured. Immunophenotyping studies of the injury site over time, quantifying infiltrating M1/M2 macrophage ratios, local IL-10 and BDNF concentrations, and circulating GA-reactive T-cell frequency, would clarify this mechanistic question and are a feasible near-term addition to the existing rat and rabbit models.

#### 7.2.3 Optimal glatiramer acetate release kinetics

The current formulation achieves a fixed concentration of glatiramer acetate in HA without specific release-rate engineering. It is unknown whether sustained slow release over weeks or a rapid initial release with subsequent decline provides optimal immunomodulatory effect in the nerve regeneration context. Future work should characterize glatiramer acetate release kinetics from the HA matrix under physiological conditions, and evaluate modified formulations (e.g., higher-degree HA, microencapsulated glatiramer acetate) for prolonged release profiles.

#### 7.2.4 AGRG behavior in ballistic and shrapnel injuries

The post-injury environment in ballistic trauma, containing metal fragments, necrotic tissue, high bacterial burden, and intense chronic inflammation, is distinct from the clean-transection model used in all current preclinical studies. Whether AGRG retains its biological activity in this more hostile environment is a clinically important question requiring dedicated preclinical investigation.

#### 7.2.5 Combination with electrical stimulation and photobiomodulation

Low-power laser irradiation was used in conjunction with the first-generation cell-loaded HA hydrogel in the early SCI experiments (48), and there is substantial evidence that brief, peri-operative electrical stimulation accelerates axonal regeneration and enhances target reinnervation in PNI models. Whether AGRG and electrical stimulation or photobiomodulation are synergistic remains an unexplored but mechanistically plausible hypothesis, and a systematic preclinical evaluation of this combination in a chronic rabbit PNI model is a feasible next experiment.

#### 7.2.6 Biomarker development for patient selection

A key gap in the current PNI literature is the absence of validated molecular biomarkers that predict regenerative capacity before surgery. Candidates include serum neurofilament light chain (NfL), a nerve-injury severity index based on pre-operative electrophysiology, and MRI fat-fraction mapping of target muscles. Incorporating such biomarker endpoints into a future AGRG clinical trial would generate data with value beyond safety and efficacy.

### 7.3 Combination strategies: Schwann cells and mesenchymal stem cells

The addition of Schwann cells or mesenchymal stem cells (MSCs) to the AGRG matrix could, in principle, further amplify the biological activity of the gel by providing a living source of neurotrophic factors, additional myelinating support, and adaptive response to the injury environment. The historical first-generation HA hydrogel loaded with embryonal spinal cord cells on microcarriers, which won the WFNS first prize in 1997, provides direct precedent for cell-enriched neurogels in SCI applications (48), and the transition from cell-loaded to purely molecular formulations was driven by translational and regulatory pragmatism, not by evidence that cellular components are unnecessary.

However, the presence of glatiramer acetate as an active immunomodulatory component within AGRG raises a genuine and, to date, unresolved safety question that we address directly here in response to Reviewer 2: does glatiramer acetate's reprogramming of the local inflammatory microenvironment impair the survival, engraftment, or differentiation of exogenously transplanted Schwann cells or MSCs?

Several considerations bear on this question, though we emphasize that none constitute direct evidence in the specific context of combined AGRG-plus-cell delivery. First, glatiramer acetate's principal local effect is a shift of the injury-site immune milieu away from a pro-inflammatory Th1/M1-dominated state and toward a pro-regenerative Th2/M2-dominated state (Section 4.4.3), and a Th2/M2-skewed environment is generally considered more, not less, favorable to the survival of exogenously transplanted cells, since M1 macrophage activity and Th1 cytokines (e.g., TNF-α, IFN-*γ*) are well-documented drivers of early graft rejection and cell death in transplantation biology generally. On this basis, the *a priori* expectation is that glatiramer acetate's immunomodulatory activity would favor, rather than impair, transplanted-cell survival. Second, the extensive multiple sclerosis clinical experience with glatiramer acetate establishes its tolerability across long-term systemic exposure in humans, though this experience does not address local co-delivery with a cellular product at a surgical site. Third, and critically, no direct experimental data currently exist on glatiramer acetate combined with Schwann cell or MSC co-delivery, either *in vitro* or *in vivo*, and we regard this absence of data as an important limitation rather than a basis for confidence. It is formally possible that local glatiramer acetate concentrations sufficient for antigliotic activity could have off-target effects on transplanted-cell viability, proliferation, or differentiation that would not be predicted from its immune-cell-directed mechanism alone; MSCs and Schwann cells express receptors and signaling machinery that could, in principle, be affected by a locally concentrated immunomodulatory peptide mixture in ways not yet characterized.

Given this uncertainty, our current program does not include AGRG-plus-Schwann-cell or AGRG-plus-MSC combination studies at this time. The near-term development plan focuses on the acellular AGRG platform, completion of the pre-IND regulatory package and a first-in-human PNI study, rather than on cell-combination work. We do not exclude Schwann cell or MSC combination strategies as a longer-term future direction: cell-enriched AGRG remains a scientifically motivated concept, particularly given the historical first-generation cell-loaded HA hydrogel precedent, and such combinations might or might not prove effective. However, in the absence of any direct experimental data on glatiramer acetate's effect on transplanted Schwann cells or MSCs, we present cell-combination strategies here as a genuinely open question rather than as a planned extension of the platform, and we make no forward-looking claim about their likely efficacy.

### 7.4 Recent clinical practice context for spinal cord injury

Contemporary clinical practice for acute traumatic SCI continues to evolve. The 2025 AO Spine Clinical Practice Recommendations for the surgical management of acute traumatic SCI provide a structured, evidence-graded framework for decompression timing, surgical technique, and perioperative management (9). These recommendations reflect meaningful progress in the surgical and systems-level management of acute SCI, but they are explicitly limited to the surgical and perioperative domain and do not include a biological therapy capable of directly countering the glial scar or promoting axonal regeneration across the lesion, the specific therapeutic gap that AGRG is intended to address (Section 1.2). The 2022 rat SCI study (2) provides proof of concept for AGRG in spinal cord applications, and a pathway toward clinical application of AGRG in SCI, where no disease-modifying biological treatment currently exists even under the most current practice recommendations, remains scientifically justified. The primary challenge is regulatory: a biological therapy for SCI would face a more complex FDA pathway than a combination product for peripheral nerve repair, requiring expanded efficacy data in a large-animal SCI model (e.g., pig) before IND submission for the SCI indication specifically. Nonetheless, the SCI application of AGRG represents a uniquely important unmet medical need, situated within the broader combinatorial and biomaterials landscape discussed in Section 6.3 (74), that justifies this expanded development investment.

Additional future opportunities include combination with next-generation, biomimetic multichannel NGCs that provide oriented microchannels mimicking native fascicular architecture (6, 7), and an AGRG-enriched processed allograft (e.g., Avance® Nerve Graft) that would combine the structural advantages of a decellularized allograft scaffold with AGRG's biological modulation, potentially extending meaningful recovery to gap lengths and injury complexities that currently exceed the performance ceiling of processed allografts alone.

## 8 Conclusion

The development arc from the first HA/embryonal-cell hydrogel of the 1990s to the current AGRG/LDP-916 formulation represents a sustained, iteratively reasoned program in translational neurosurgical research, in which each generation of the technology was designed to address a specific biological deficiency identified in the previous iteration.

The central insight that drives this evolution, and that distinguishes the AGRG paradigm from conventional nerve conduit technology, is that structural bridging of a nerve gap is necessary but not sufficient. The inhibitory post-injury microenvironment, dominated by reactive gliosis and CSPG accumulation, does not merely create a passive obstacle to axonal advance: it actively repels and collapses growth cones through receptor-mediated signaling cascades, oxidative membrane damage, and macrophage/microglial pro-inflammatory amplification. AGRG addresses both dimensions simultaneously, providing structural guidance through the laminin peptide and HA scaffold, and actively modulating the injury microenvironment through glatiramer acetate's immunomodulatory and antigliotic activity and DL-α-tocopherol's antioxidant protection.

AGRG thereby exemplifies the “neurogel” paradigm articulated by Rochkind (5): a bioactive hydrogel platform designed not merely to bridge a gap but to create a regenerative niche within which axons can grow, Schwann cells can migrate and myelinate, and the injured nerve can approach functional recovery. The preclinical data summarized in this review, from *in vitro* neurite outgrowth, to a dose-finding rat study, to a chronic rabbit 25 mm-gap model, to rat acute and chronic SCI, support the conclusion that this approach works at multiple biological levels, in multiple models, and across both peripheral and central nervous system applications, while several mechanistic and safety questions identified in Section 7 remain to be resolved.

The translational path forward is concrete and near-term but not yet complete: Maxonis Ltd. is compiling the nonclinical data package required for a pre-IND interaction with the FDA (Section 7.1), a milestone that, if the interaction confirms the adequacy of the existing nonclinical package or clarifies the specific additional studies required, would position AGRG for IND filing and a first-in-human PNI study. Whether AGRG will ultimately prove to be a viable alternative to autologous nerve grafting, one that matches or exceeds the gold standard without donor-site morbidity, and that can address the chronic, complex, long-gap injuries that remain beyond the reach of current technology, will be determined by that prospective clinical evidence, not by the preclinical data alone.

Several important caveats deserve explicit acknowledgment. All current AGRG efficacy data are derived from animal models, and species differences in nerve regeneration biology mean that human outcomes cannot be assumed to replicate animal results. The rabbit chronic PNI model and the rat sciatic critical-gap model, while more stringent than many published NGC studies, involve mechanically clean transection injuries in healthy young animals, conditions that do not fully replicate the infected, scarred, ballistic, or metabolically compromised injuries encountered in clinical practice. The nine-week denervation delay in the rabbit model approximates chronic PNI but does not approach the multi-year denervation seen in some clinical presentations. As detailed in Sections 5.3, 5.5, and 7.2–7.3, the relative contribution of glatiramer acetate vs. the concurrent antioxidant substitution, the local-vs.-systemic mechanism of glatiramer acetate action, and the safety of glatiramer acetate in combination with exogenous cell therapy all remain open scientific questions rather than settled conclusions. These limitations do not undermine the value of the preclinical data, but they define the specific evidentiary gaps that future nonclinical studies, and, ultimately, a first-in-human trial, will need to close.

From a scientific perspective, the AGRG program has advanced the conceptual framework for nerve repair technology beyond the purely structural paradigm toward a biological modulation paradigm, situated within a broader field, described in Section 6.3, that is converging on the same principle from multiple directions (biomaterials, cell therapy, growth factor delivery, and combinatorial strategies) (74). The evidence that a carefully formulated, regulatory-tractable molecular gel can approach or exceed autograft performance in rigorous animal models challenges the assumption that biological complexity is irreplaceable, and opens a broader design space for next-generation nerve repair innovations.

This review is dedicated to the memory of Professor Zvi Nevo, whose extraordinary scientific breadth, spanning proteoglycan biochemistry, ECM biology, and translational neuroscience, provided the intellectual foundation for every generation of the GRG/AGRG technology, and whose collaborative vision sustained this research program across more than two decades.
